# Microcystin-Degrading Activity of an Indigenous Bacterial Strain *Stenotrophomonas acidaminiphila* MC-LTH2 Isolated from Lake Taihu

**DOI:** 10.1371/journal.pone.0086216

**Published:** 2014-01-09

**Authors:** Fei Yang, Yuanlong Zhou, Lihong Yin, Guangcan Zhu, Geyu Liang, Yuepu Pu

**Affiliations:** Key Laboratory of Environmental Medicine Engineering, Ministry of Education, School of Public Health, Southeast University, Nanjing, China; University of New South Wales, Australia

## Abstract

Microcystin-LR (MC-LR) and microcystin-RR (MC-RR) produced by harmful cyanobacterial blooms (HCBs) pose substantial threats to the ecosystem and public health due to their potential hepatotoxicity. Degradation of microcystins (MCs) by indigenous bacteria represents a promising method for removing MCs from fresh water without harming the aquatic environment, but only a few microcystin (MC)-degrading bacteria have been isolated and had their mechanisms reported. This study aimed to isolate indigenous bacteria from Lake Taihu, and investigate the capability and mechanism of MC degradation by these bacteria. During a *Microcystis* bloom, an indigenous MC-degrading bacterium designated MC-LTH2 was successfully isolated from Lake Taihu, and identified as *Stenotrophomonas acidaminiphila* based on phylogenetic analysis. In the presence of MC-LR together with MC-RR, the strain MC-LTH2 was capable of totally degrading both simultaneously in 8 days, at rates of 3.0 mg/(L⋅d) and 5.6 mg/(L⋅d), respectively. The degradation rates of MCs were dependent on temperature, pH, and initial MC concentration. Adda (3-amino-9-methoxy-2, 6, 8-trimethyl-10-phenyldeca-4, 6-dienoic acid) was detected as an intermediate degradation product of MCs using high performance liquid chromatography coupled with time-of-flight mass spectrometry (HPLC-TOF-MS). To the best of our knowledge, this is the first report of *Stenotrophomonas acidaminiphila* capable of degrading two MC analogues and other compounds containing Adda residue completely under various conditions, although the *mlrA* gene in the strain was not detected. These results indicate the *Stenotrophomonas acidaminiphila* strain MC-LTH2 possesses a significant potential to be used in bioremediation of water bodies contaminated by MC-LR and MC-RR, and is potentially involved in the degradation of MCs during the disappearance of the HCBs in Lake Taihu.

## Introduction

Harmful cyanobacterial blooms (HCBs) have occurred frequently in eutrophic lakes all over the world in the past two decades, producing a series of cyanotoxins, such as microcystins (MCs), nodularins and saxitoxins [1], [2]. MCs are predominantly produced by freshwater cyanobacteria including species of *Microcystis*, *Anabaena*, *Oscillatoria*, and *Nostoc* [2], [3]. More than 90 analogues of MCs have been identified until now [4], [5]. They have the same genetic structure cyclo-(D-Ala-X-D-MeAsp-Z-Adda-DGlu-Mdha-), where X and Z represent variable L-amino acids, and Adda refers to the b-amino acid residue of 3-amino-9-methoxy-2,6,8-trimethyl-10-phenyldeca-4,6-dienoic acid [6], [7], [8]. Most MCs are extremely toxic to mammals, fish, plants and invertebrates by inhibition of protein phosphatases [2], [3], [9]. Microcystin-LR (MC-LR) and microcystin-RR (MC-RR) are the two most common MCs, and the presence of the two kinds of MCs in water, especially in drinking water, is of great concern, since chronic exposure to low concentrations of MCs may promote tumor growth [2], [4], [10]. Thus, the World Health Organization (WHO) recommends that MC-LR concentration in drinking water should not exceed 1 µg/L [11].

MCs are very stable in the environment because of their cyclic structure, and conventional drinking water treatments have limited efficacy in removing dissolved MCs [4], [12]. However, MCs can be readily degraded by a range of aquatic bacteria [13]. Degradation of MCs by indigenous bacteria represents a promising method for removing MCs from fresh water without harming the aquatic environment, but only a few microcystin (MC)-degrading bacteria have been isolated and had their mechanisms reported [12], [14], [15]. So far most of the isolated MC-degrading bacteria appear to be limited to the family *Sphingomonadaceae* [8], [14], [16]. To date, a single gene cluster (*mlrA*, *mlrB*, *mlrC* and *mlrD*) responsible for the degradation of MC-LR in *Sphingomonas* sp. ACM-3962 was identified by Bourne, et al (2001), and the most important gene *mlrA* encoded an enzyme responsible for the hydrolytic cleavage of the cyclic MC-LR [3], [17].

Lake Taihu is the third-largest lake in China, and is essential to millions of people for drinking water, recreation, aquaculture, and industrial activities. During the last three decades, Lake Taihu has experienced HCBs every year [18], [19], [20], [21]. In 2007, the HCBs coverage area incredibly reached 40% of the total area of Lake Taihu, which resulted in more than 2 million residents of the city of Wuxi being without drinking water for a week [22], [23], [24]. It’s desirable to isolate and identify bacteria capable of MC degradation, since removing MC-LR and MC-RR from water supplies is very important. In this study, an indigenous bacterium capable of degrading MC-LR and MC-RR simultaneously was isolated from Lake Taihu during a *Microcystis* bloom, and the capability and mechanism of MC degradation by this strain were investigated.

## Materials and Methods

### Ethics Statement

Sampling from cyanobacteria-salvage yards in Lake Taihu doesn’t require specific permission, because Lake Taihu is a public lake belonging to the Chinese central government, providing 40 million people with recreation, drinking water, aquaculture, and industrial use. It has a total water surface area of about 2338 km^2^, an average water depth of 1.89 m and a water volume of approximately 4.43×10^12^ L. Since the HCBs have occurred frequently in Lake Taihu during the last few decades, the Chinese central government has paid considerable attention to it and the Ministry of Science and Technology of China has granted us some large projects on cyanobacteria control and MC degradation in Lake Taihu (e.g. The National Natural Science Foundation of China (30972440), the National Science and Technology Major Project (2012ZX07101-005, 2012ZX07403-001)). Moreover, sampling from Lake Taihu did not involve endangered or protected species, since sludge samples were collected using sterilized (autoclaved at 121°C for 20 min) glass tubes.

### Reagents

MC-LR and MC-RR were extracted from lyophilized *Microcystis aeruginosa* cells in methanol and water under conditions previously reported by Hu et al (2009). The lyophilized *M. aeruginosa* cells were purchased from the Fresh Algae Culture Collection of the Institute of Hydrobiology, Chinese Academy of Sciences. The mineral salt medium (MSM) used for the bacterial isolation and degradation contained (g/L) MgSO_4_⋅7H_2_O 1.0; KH_2_PO_4_ 0.5; K_2_HPO_4_ 4.0; NaCl 1.0; CaCl_2_ 0.02; FeSO_4_ 0.005; MnCl_2_⋅4H_2_O 0.005; ZnCl_2_ 0.005; CuCl_2_ 0.0005 [20]. Methanol for high performance liquid chromatography coupled with time-of-flight mass spectrometry (HPLC-TOF-MS) analysis was purchased from Dikma Technology Incorporation located in the USA.

### Isolation and Identification of MC-degrading Bacteria

A sludge sample was collected from cyanobacteria-salvage yards in Lake Taihu, China. The sludge (10 g wet weight) was suspended in sterile (autoclaved at 121°C for 20 min) Taihu Lake water (90 mL) by shaking at a constant temperature (30°C, 120 rpm) for 30 min. After standing for 15 min, 5 mL of the supernatant was inoculated into 45 mL of MSM containing MC-LR (21.2 mg/L) and MC-RR (39.2 mg/L). After shaking at a constant temperature (30°C, 120 rpm) for six days, 5 mL of the resulting solution were inoculated into 45 mL of MSM with MCs to form a new subculture. The fourth subculture was spread onto the MSM agar plates (MSM; 2% agar), which contained MC-LR (21.2 mg/L) and MC-RR (39.2 mg/L). Single colonies from MSM agar plates were transferred to MSM containing MC-LR and MC-RR, and the concentrations of the remaining MCs were monitored using HPLC. One bacterial strain with high MC-degrading activities was obtained and designated as MC-LTH2.

The universal primers (sense: 5′-AGAGTTTGATCMTGGCTCAG-3′; antisense: 5′-TACGGYTACCTTGTTACGACTT-3′) were used in PCR amplification of the 16S rDNA fragment of MC-LTH2 using conditions described previously [19]. The PCR products were sequenced by the BGI Company located in China. Comparisons of nucleotide sequences were performed using the National Center for Biotechnology Information database (http://www.ncbi.nlm.nih.gov/BLAST). Similar 16S rDNA sequences were downloaded from Genbank and all the sequences were aligned using the program ClustalW 2.1. A phylogenetic tree was constructed using the neighbor-joining method based on the software MEGA 4 [25], and the tree was then tested with the bootstrap procedure using 1000 random samples.

### Biodegradation of MCs

The strain MC-LTH2 was cultured for 48 h with shaking at a constant temperature (30°C, 120 rpm) in MSM containing MC-LR (21.2 mg/L) and MC-RR (39.2 mg/L). The bacterial cells were harvested by centrifugation (5000×g, 15 min, 4°C), washed twice with 0.02 M sodium phosphate buffer (pH = 7.2), suspended into MSM containing MCs, and then cultured under different incubation circumstances including different temperature (4, 20, 30, and 37°C), initial MC concentrations (5.0, 12.1, and 21.2 mg/L for MC-LR, and 8.2, 20.7 and 39.2 mg/L for MC-RR), and pH values (pH = 6, 7, 8). In every experiment, 300 µL samples for MC analysis were withdrawn at regular intervals, and the concentrations of MCs in the samples were then detected using HPLC after centrifugation (12000×g, 15 min, 4°C). All experiments were carried out in duplicate and bacterial-free medium was employed as a control.

### Genetic and Chemical Analysis of Bacterial Isolates and their Degradation Products

MCs and their degradation products were monitored using an Agilent 1100 HPLC machine with a Zorbax Extend C_18_ column (4.6×150 mm, 5 µm, Agilent, USA) and a variable wavelength detector (VWD) set at 238 nm. The mobile phase was a mixture of methanol and 0.1% trifluoroacetic acid aqueous solution (55∶45, v/v) set at a flow rate of 1 mL/min. The column temperature was maintained at 40°C and the injection volume was 20 µL [19], [20].

The main degradation products of MCs were identified by an HPLC coupled with an Agilent 6224 TOF-MS with a mass range from m/z 200 to 800 in positive electrospray ionization (ESI) mode. The temperature of the analytical column (Zorbax Extend C_18_, 2.1×50 mm, 1.8 µm, Agilent, USA) was maintained at 40°C, and the injection volume was 2 µL. The ESI-TOF-MS parameters were set as follows: capillary voltage, 3500 V; flow of drying gas, 9 L/min; gas temperature, 325°C; fragmentor voltage, 150 V; nebulizer pressure, 40 psi.

Two specific oligonucleotide primers (sense: 5′-GACCCGATGTTCAAGATACT-3′; antisense: 5′-CTCCTCCCACAAATCAGGAC-3′) purchased from the BGI company were used in PCR to screen bacterial isolates for the *mlrA* gene [26]. The PCR ran with an initial denaturation at 95°C for 5 min, 35 thermal cycles of denaturation for 10 seconds at 98°C, annealing for 30 seconds at 56°C, extension for 30 seconds at 72°C and a final elongation for 5 min at 72°C. A positive control (*Bordetella* sp. strain MC-LTH1, accession number: KC734882.1) for the *mlrA* gene was used.

## Results and Discussion

### Isolation and Identification of MC-degrading Bacteria

**Figure 1:**
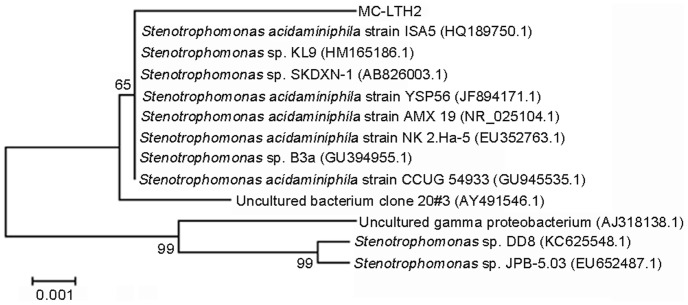
A phylogenetic tree based on 16S rDNA sequences of MC-LTH2 and other closely related species. The numbers at the nodes are the levels of bootstrap support (%) based on the neighbor-joining analyses of 1,000 resampled datasets. The scale bar represents 0.001 nucleotide substitutions per position.

A bacterium designated MC-LTH2 capable of degrading MC-LR and MC-RR simultaneously was isolated from Lake Taihu, China. The bacterial strain was aerobic, gram-negative. Analysis of its 16S rDNA sequence revealed that the bacterium belonged to the species *Stenotrophomonas acidaminiphila* (*S. acidaminiphila*) (Figure 1). The BLAST search available on the NCBI web showed that the 16S rDNA sequence of strain MC-LTH2 was most similar to *S. acidaminiphila* strain CCUG 54933 (99% similarity, accession number: GU945535.1). The nucleotide sequence of 16S rDNA from MC-LTH2 was deposited in the NCBI database with accession number KF305533.

Simultaneous degradation of MC-LR and MC-RR by *S. acidaminiphila* has not been reported before, although MC degradation has been addressed for a few different bacterial strains including *Sphingomonas* sp. [3], [27], [28], *Pseudomonas* sp. [29], *Arthrobacter*, *Brevibacterium* and *Rhodococcus* sp. [30], *Methylobacillus* sp. [7], [8], *Sphingopyxis* sp. [31], [32], and *Bacillus* sp. [16]. The similarity of *Stenotrophomonas* sp. EMS to *Stenotrophomonas maltophila* [20] also means that it belongs to a different *Stenotrophomonas* strain to MC-LTH2.

### Biodegradation of MCs in Batch Experiments

**Figure 2:**
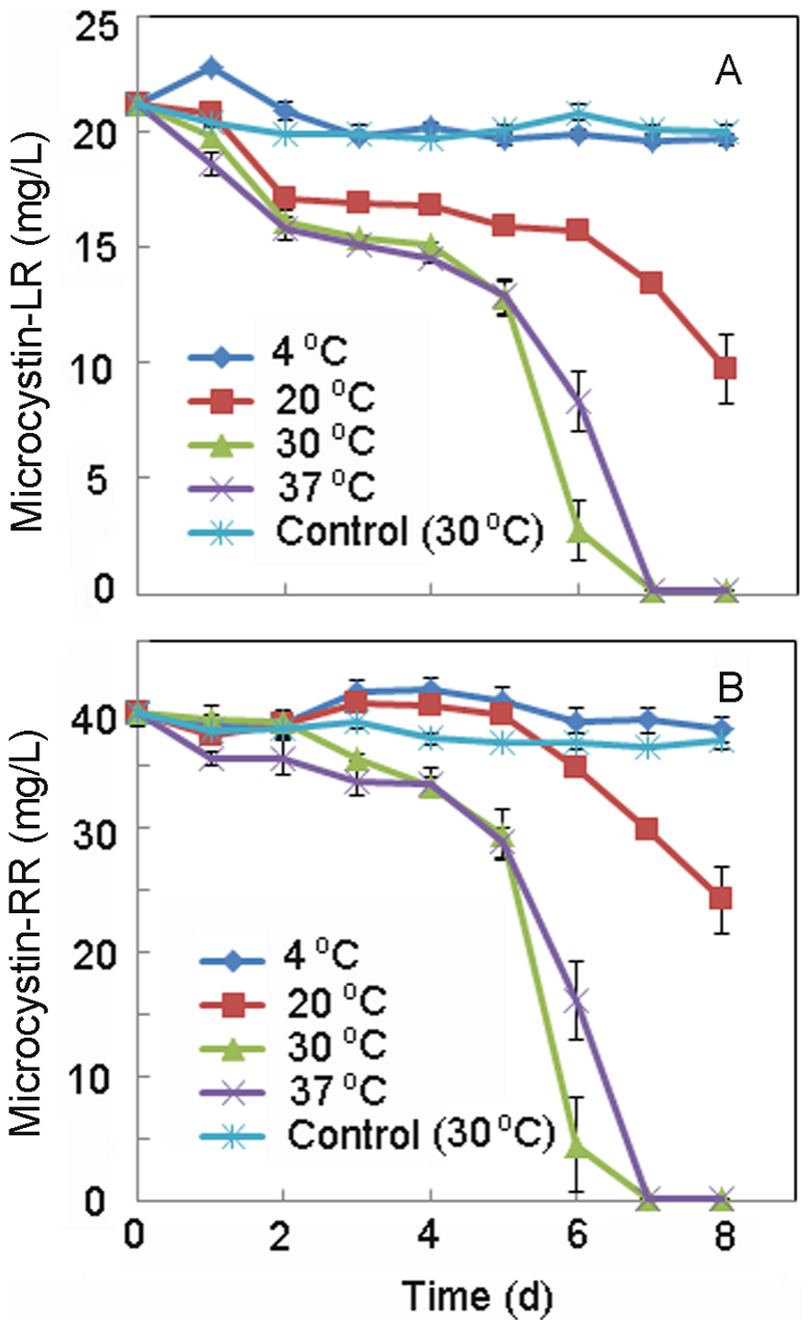
Effect of incubation temperature on the degradation of MC-LR (A) and MC-RR (B) by MC-LTH2.

**Figure 3:**
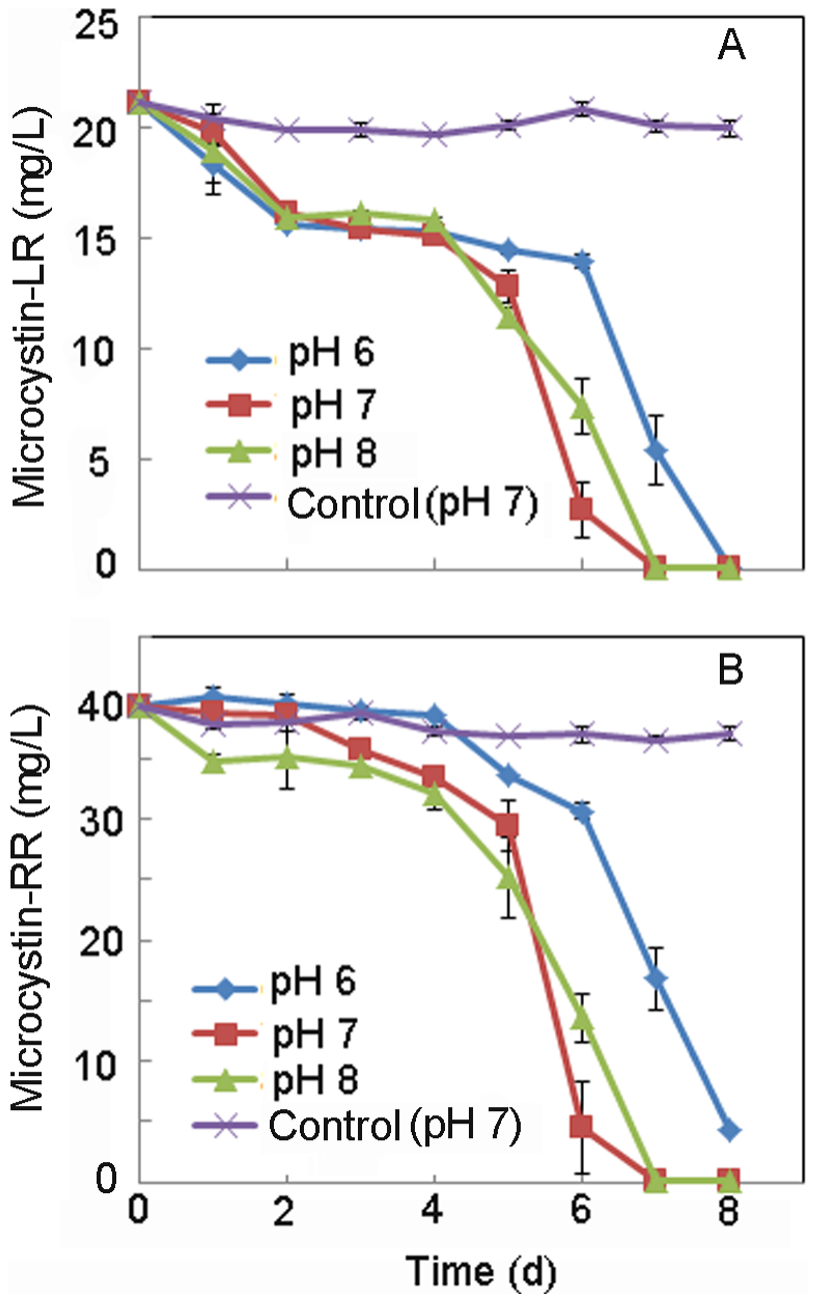
Effect of pH values on the degradation of MC-LR (A) and MC-RR (B) by MC-LTH2.

**Figure 4:**
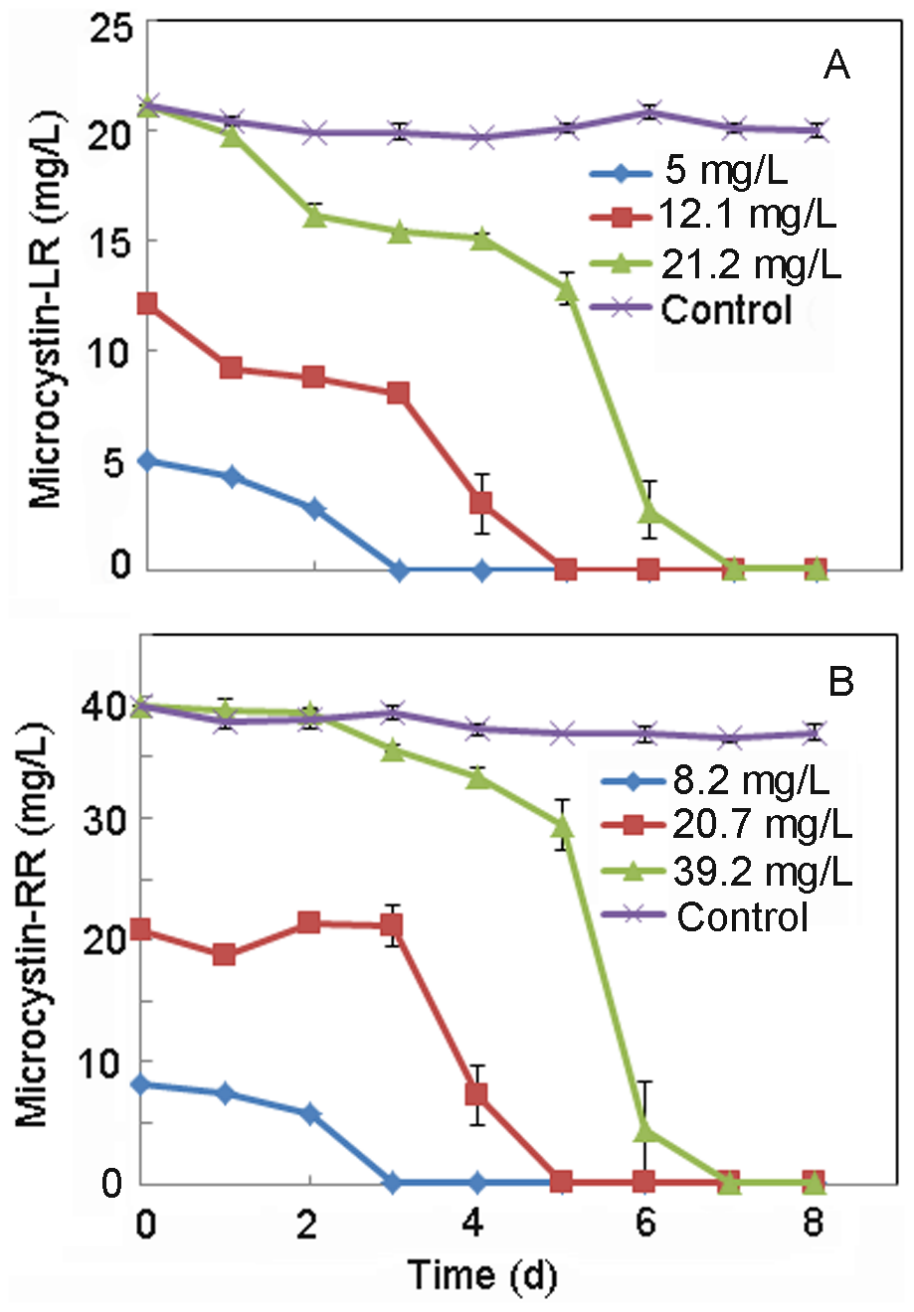
Effect of initial microcystin concentration on the degradation of MC-LR (A) and MC-RR (B) by MC-LTH2.

MC-LR and MC-RR were efficiently degraded simultaneously by the bacterium MC-LTH2 (Figure 2, 3, and 4). The degradation rates were strongly dependent on the incubation temperature (Figure 2). The highest degradation rate of MC-LR was 3.0 mg/(L⋅d) (Figure 2A) and that of MC-RR was 5.6 mg/(L⋅d) (Figure 2B), which occurred at 30°C. The impact of temperature on the rate of MC degradation was almost the same for MC-LR and MC-RR. The degradation rate of MCs increased with temperature from 4 to 30°C. The control showed no degradation of MC-LR and MC-RR after incubation for 8 days.

The two MC analogues were efficiently removed simultaneously at near neutral pH although the optimum pH for degradation was neutral or weak alkaline conditions (Figure 3). The degradation rate of MC-LR and MC-RR by the strain MC-LTH2 varied with different initial concentrations (Figure 4). At the highest initial MC concentrations (21.2 mg/L for MC-LR, and 39.2 mg/L for MC-RR), MC-LR and MC-RR were simultaneously degraded at the highest rates of 3.0 mg/(L⋅d) and 5.6 mg/(L⋅d), respectively. A lag period of 4 d was obvious prior to isolate MC-LTH2 initiating degradation of both analogues, with complete removal observed by day 7. After a shorter lag period of 3 d, 12.1 mg/L MC-LR and 20.7 mg/L MC-LR were simultaneously degraded at the rates of 2.4 mg/(L⋅d) and 4.1 mg/(L⋅d). There was no apparent lag period when the lowest MC concentrations (MC-LR ∼5 mg/L, and MC-RR ∼8.2 mg/L) were degraded at the lowest rates of 1.6 mg/(L⋅d) and 2.8 mg/(L⋅d), respectively. Thus, the average MC degradation rates and the lag period increased with initial MCs concentration.

It is important to examine the effect of pH on the degradation activity of MC-degrading bacteria, since the pH of most water bodies changes dynamically during the occurrence of HCBs [33], [34]. The MC-LTH2 strain could effectively degrade MCs in weak acid conditions, and preferred to degrade MCs in neutral and weak alkaline conditions (Figure 3). These results showed that the MC-LTH2 strain might be actively involved in the dramatic degradation of MCs during the HCBs in Lake Taihu.

The highest degradation rate of MC-LR by MC-LTH2 (3.0 mg/(L⋅d)) was higher than that by *Sphingomonas* ACM-3962 (1.7 mg/(L⋅d)) and LH21 (2.1 mg/(L⋅d)) [14]. Moreover, the strain MC-LTH2 had a slightly higher MC-RR degradation rate (2.8 mg/(L⋅d)) at the initial concentration 8.2 mg/L) (Figure 4B) compared to a MC-RR degradation rate by *Bacillus* sp. SSZ01 (2.5 mg/(L⋅d) at initial concentration 10 mg/L) [16]. Although the simultaneous degradation rates of MC-LR and MC-RR by MC-LTH2 were lower than those by *Sphingomonas* Y2 (13 mg/(L⋅d) for MC-LR and 5.4 mg/(L⋅d) for MC-RR) [1], they are higher than those by *Stenotrophomonas* sp. EMS (0.7 mg/(L⋅d) for MC-LR and 1.7 mg/(L⋅d) for MC-RR) [20]. The different bacterial species used in these studies may explain why they have different MC degradation rates. A lag phase existed prior to the MC-LTH2 initiation of degradation for both of the MC analogues in this study. It is not clear why this lag phase existed. A possible explanation for the lag phase is that the synthesis of MC degradative enzymes (microcystinase) was repressed by some catabolite repressor substrates present in the *M. aeruginosa* extracts [13]. In this study, more catabolite repressor substrates may have existed in MSM when spiked with the higher initial concentrations of MCs, which could have resulted in the extended lag period prior to the onset of degradation.

### Analysis of MCs and their Degradation Products and Detection of the *mlrA* Gene

**Figure 5:**
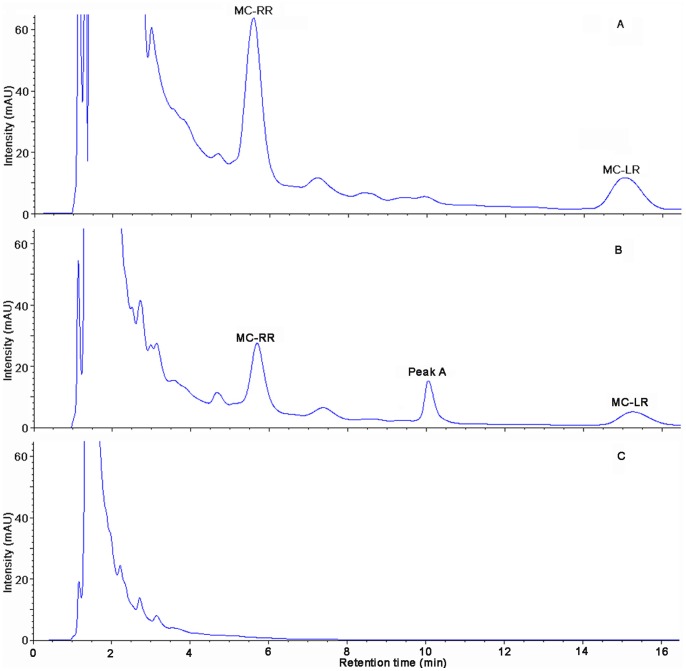
HPLC chromatograms obtained during the biodegradation of MCs. At time zero (A), 6 d (B), 8 d (C). Peaks A showed an intermediate product of MCs.

**Figure 6:**
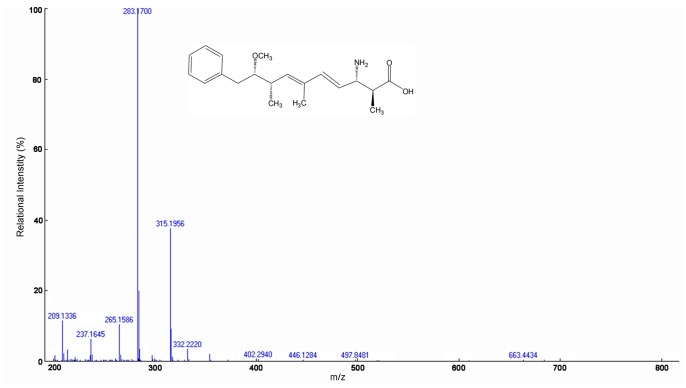
ESI-TOF-MS spectrum and putative structure of the biodegradation product of MC-LR and MC-RR.

HPLC chromatograms of MCs and their degradation products were obtained (Figure 5). The peak area of MC-LR (initial concentration of 21.2 mg/L) and MC-RR (39.2 mg/L) decreased significantly after incubation (30°C, 120 rpm) and a main intermediate degradation product of MCs (peak A) was apparent in 6 d (Figure 5B). The disappearance of all the peaks demonstrated the complete degradation of MC-LR and MC-RR by MC-LTH2 (Figure 5C). The degradation product peak A was further identified using the HPLC-TOF-MS system, and exhibited several accompanying ions at m/z 663.4434, 332.2220, 315.1956, 283.1700, and 265.1586 (Figure 6). Two of them, at m/z 663.4434 and m/z 332.2220, were a dimeric ion [2M+H]^+^ and a protonated ion ([M+H]^+^), respectively. In addition, three of them, at m/z 315.1956 (consistent with the loss of an ammonia [M+H−NH_3_]^+^), 283.1700 and 265.1586 (further loss of a methanol [M+H−NH_3_−MeOH]^+^ and a water [M+H−NH_3_−MeOH−H_2_O]^+^), were fragment ions. These ions were identical to the Adda values addressed from the final degradation products of the *Sphingomonas* sp. B-9 and *Sphingopyxis* C-1 [31], [35].

Similar HPLC chromatograms showing Adda as an MC degradation product were previously reported by Imanishi, et al (2005), so this degradation process was predicted to be comparable to that by *Sphingomonas* sp. B-9 and ACM-3962 [3], [35]. The bacteria *Sphingomonas* sp. B-9 and ACM-3962 degraded MC-LR because they contained the *mlrA* gene, which encoded a very important hydrolytic enzyme MlrA capable of initiating MCs degradation [17], [35]. The *mlrA* gene has been characterized and been successfully used to detect the presence of MC-degrading bacteria [17], [26], [36]. To investigate whether the mechanism of MC degradation by the stain MC-LTH2 was similar to that by *Sphingomonas* sp. ACM-3962, DNA extracted from MC-LTH2 and specific oligonucleotide primers were used for PCR targeting the *mlrA* gene with conditions as described previously [26]. The amplification product for *mlrA* was not obtained, whereas the *mlrA* homologue was observed in the positive control. It is possible that the indigenous MC-degrading bacterium MC-LTH2 from Lake Taihu does not contain *mlrA* homologues or that there are great differences in the sequences [30], [36]. Interestingly, the results of the PCR also suggested that the mechanism of MCs degradation by *S. acidaminiphila* MC-LTH2 was different to that by *Stenotrophomonas* sp. strain EMS containing an *mlrA* homologue [20], although both bacterial strains belonged to *Stenotrophomonas* species. To elucidate the mechanism of MCs degradation by MC-LTH2, experiments have been in progress to identify the MC-degrading enzyme(s) and the genes involved in MC degradation in the strain MC-LTH2.

The ability of MC-LTH2 to efficiently degrade MC-LR and MC-RR simultaneously is demonstrated by all substances containing the Adda residue (critical part for MC toxicity) [28] being degraded by day 8 (Figure 5C). These results confirmed that the MC-LTH2 strain might be actively involved in the dramatic degradation of MCs during the disappearance of the HCBs in Lake Taihu. Further study is needed to evaluate the applicability of this strain for large scale use in various types of water bodies contaminated with MCs. Since the application of a biological sand filter embeded with MC-degrading bacteria (e.g., *Sphingomonas* sp. MJ-PV, *Sphingopyxis* sp. LH21) removed MC-LR and MC-LA from source water successfully [12], [14], an effective in-situ degradation of MC-LR and MC-RR might be achieved using a biofilter with immobilized MC-LTH2 in the future.

## Conclusion

An indigenous MC-degrading bacterium MC-LTH2 isolated from Lake Taihu was identified as *S. acidaminiphila* based on 16S rDNA sequence analysis. This is the first report of *S. acidaminiphila* being capable of degrading MC-LR and MC-RR simultaneously. The *S. acidaminiphila* strain MC-LTH2 degraded MCs at different rates, and the degradation rates were dependent on temperature, pH, and initial MC concentration. The strain MC-LTH2 also completely degraded other compounds containing the Adda residue besides MCs under various conditions, although the *mlrA* gene in the strain was not detected. Therefore, *S. acidaminiphila* strain MC-LTH2 possesses a significant potential to be used in bioremediation of water bodies contaminated by MC-LR and MC-RR, and is potentially involved in the degradation of MCs during the disappearance of the HCBs.
